# Supplementary figures and images for: Per-sample immunoglobulin germline inference from B cell receptor deep sequencing data
Source: PLoS Comput Biol. 2019 Jul 22;15(7):e1007133. doi: 10.1371/journal.pcbi.1007133 (PMC6675132; doi:10.1371/journal.pcbi.1007133)

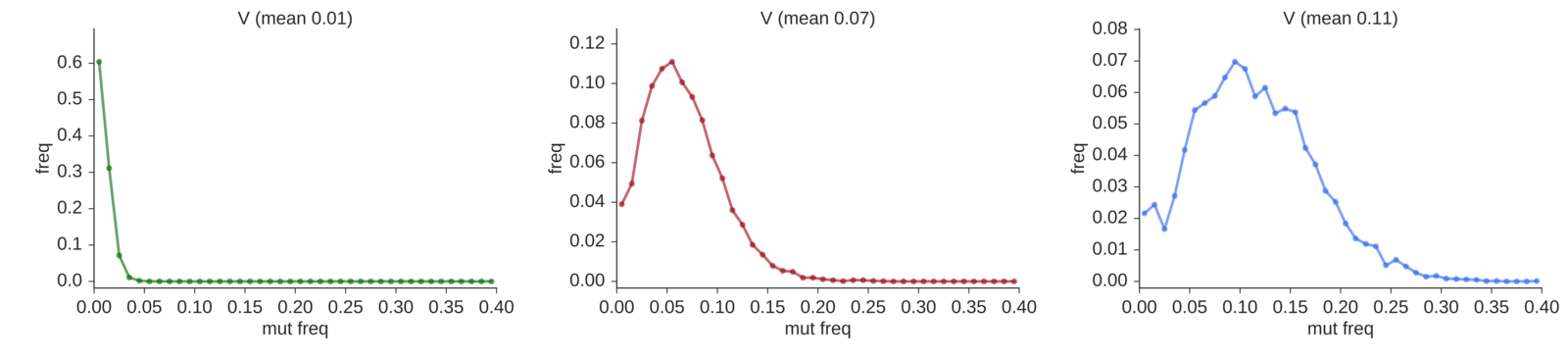

Supplement: S1 Fig — In the context of these distributions, the full-repertoire samples (Figs 3, 4, 5 and 6) correspond to a mean value of 0.02 (“low-SHM” samples) and 0.06 (“high-SHM” samples). (TIFF) [file pcbi.1007133.s001.tiff]

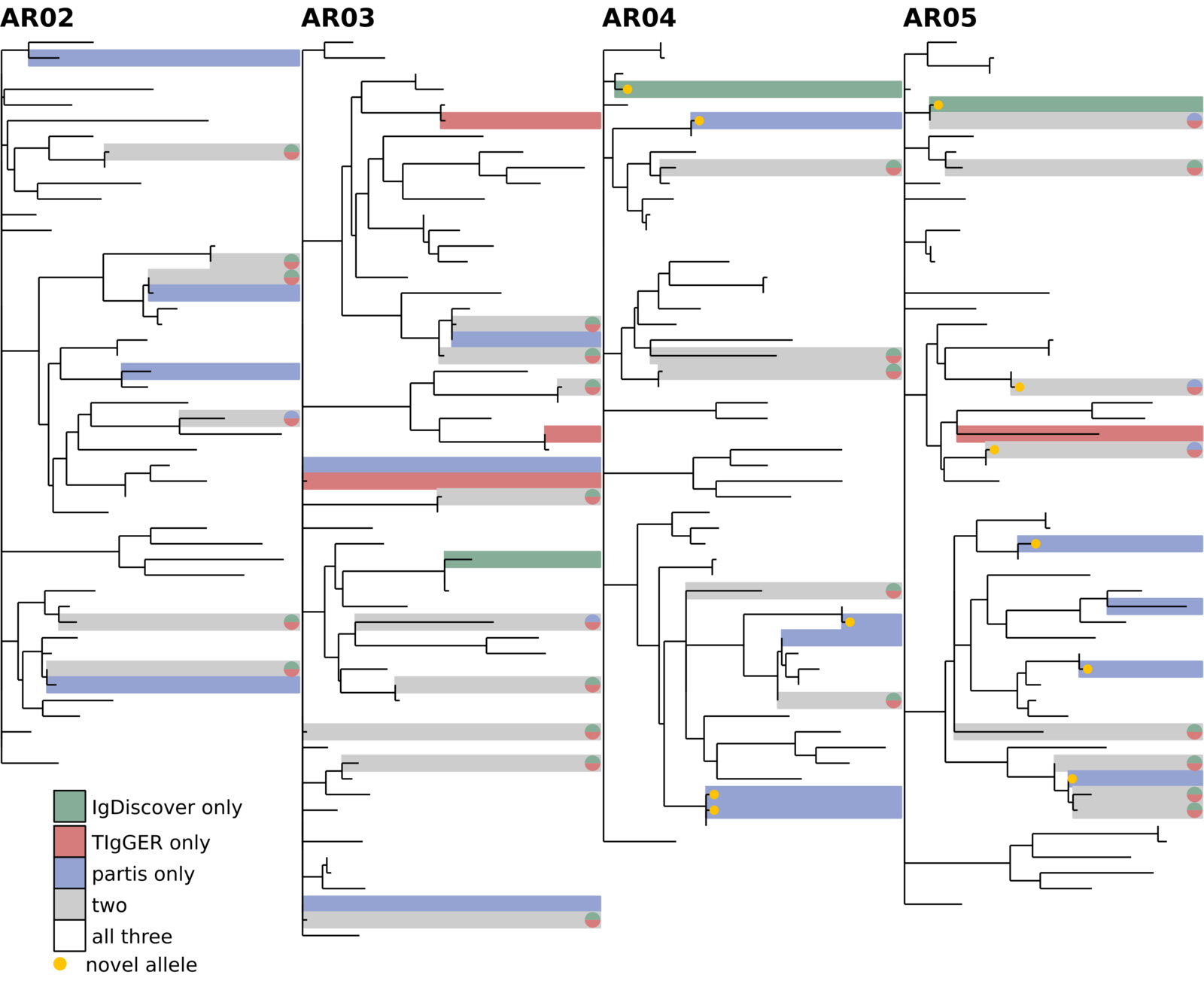

Supplement: S2 Fig — (TIFF) [file pcbi.1007133.s002.tiff]

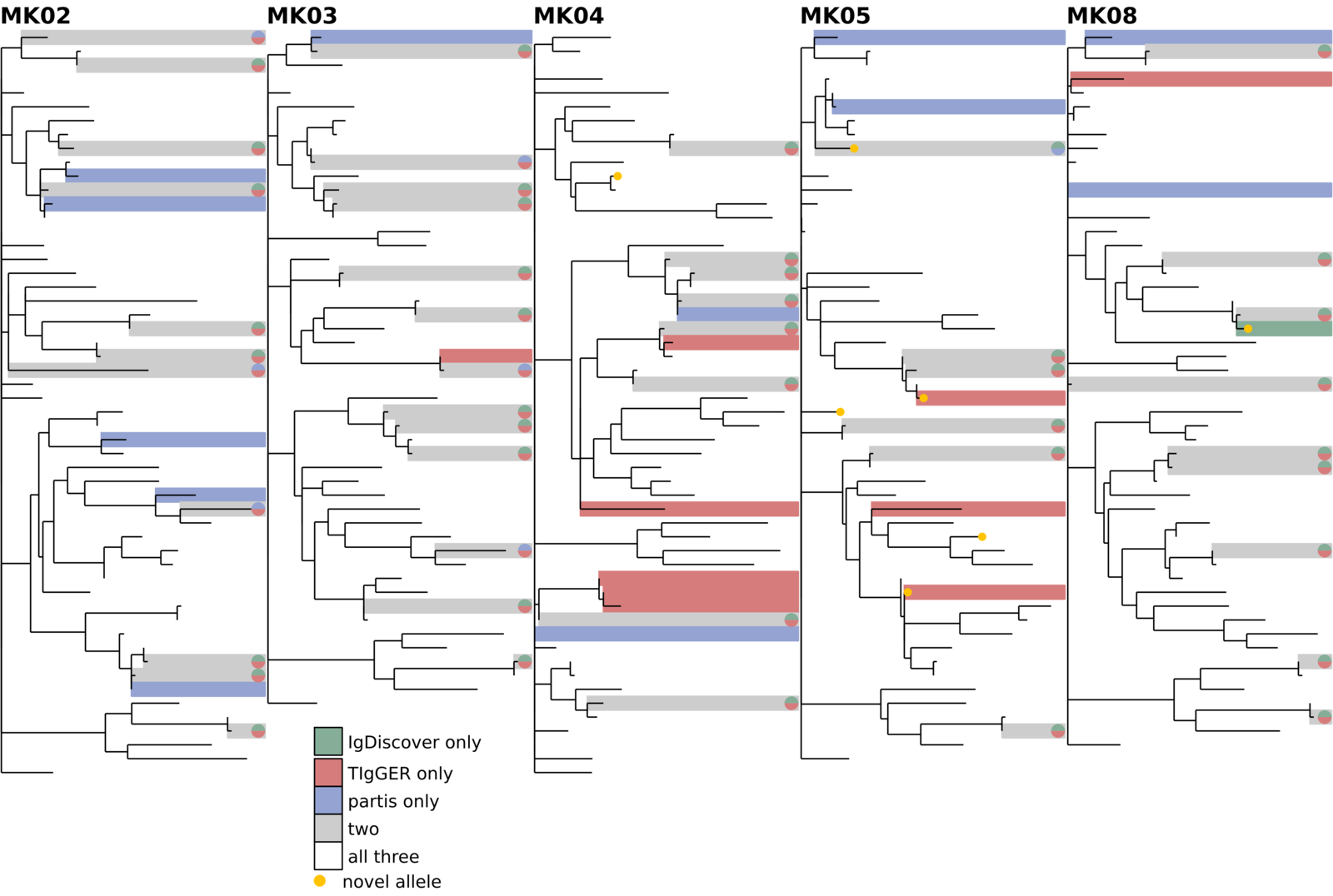

Supplement: S3 Fig — (TIFF) [file pcbi.1007133.s003.tiff]

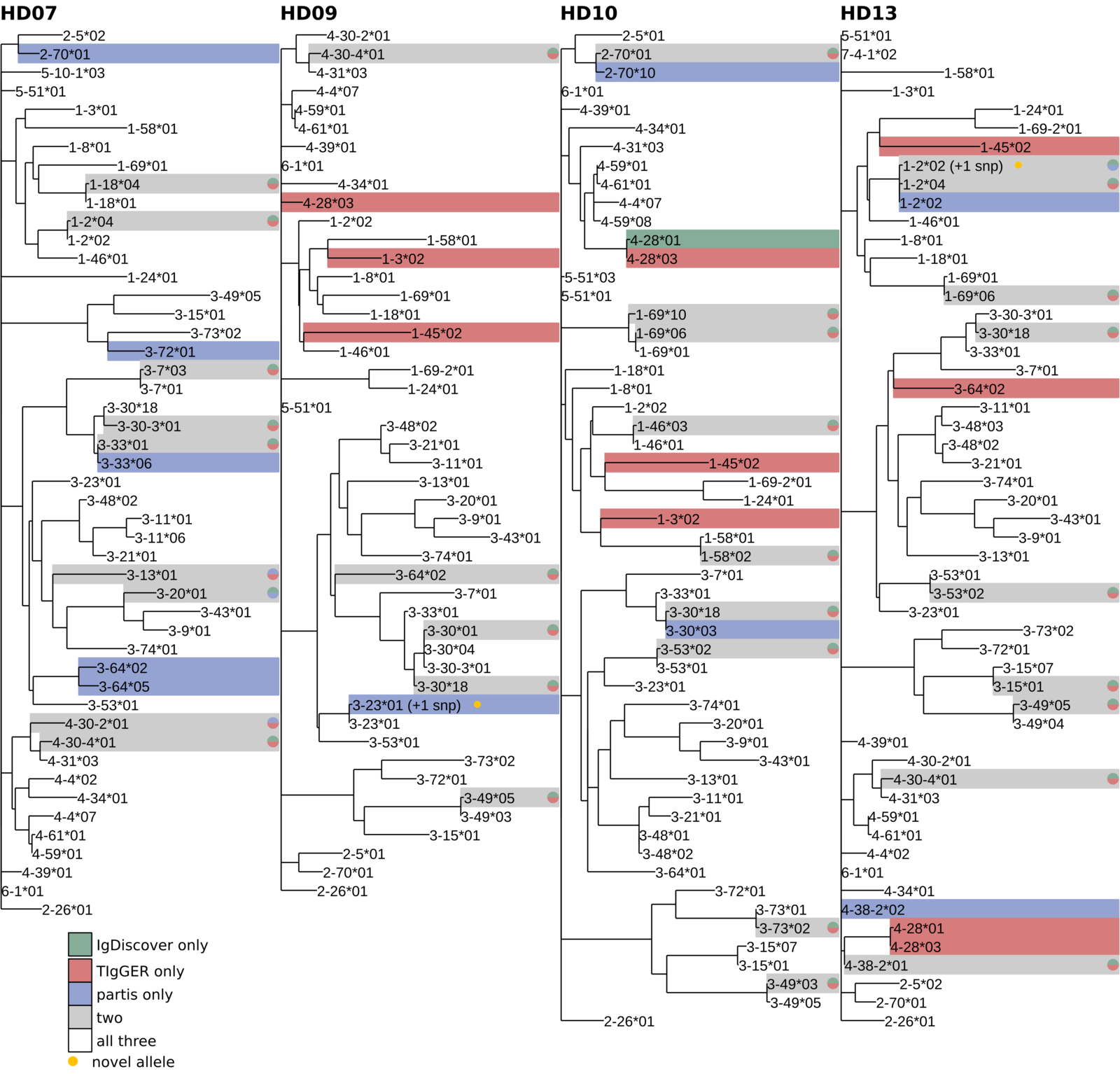

Supplement: S4 Fig — (TIFF) [file pcbi.1007133.s004.tiff]

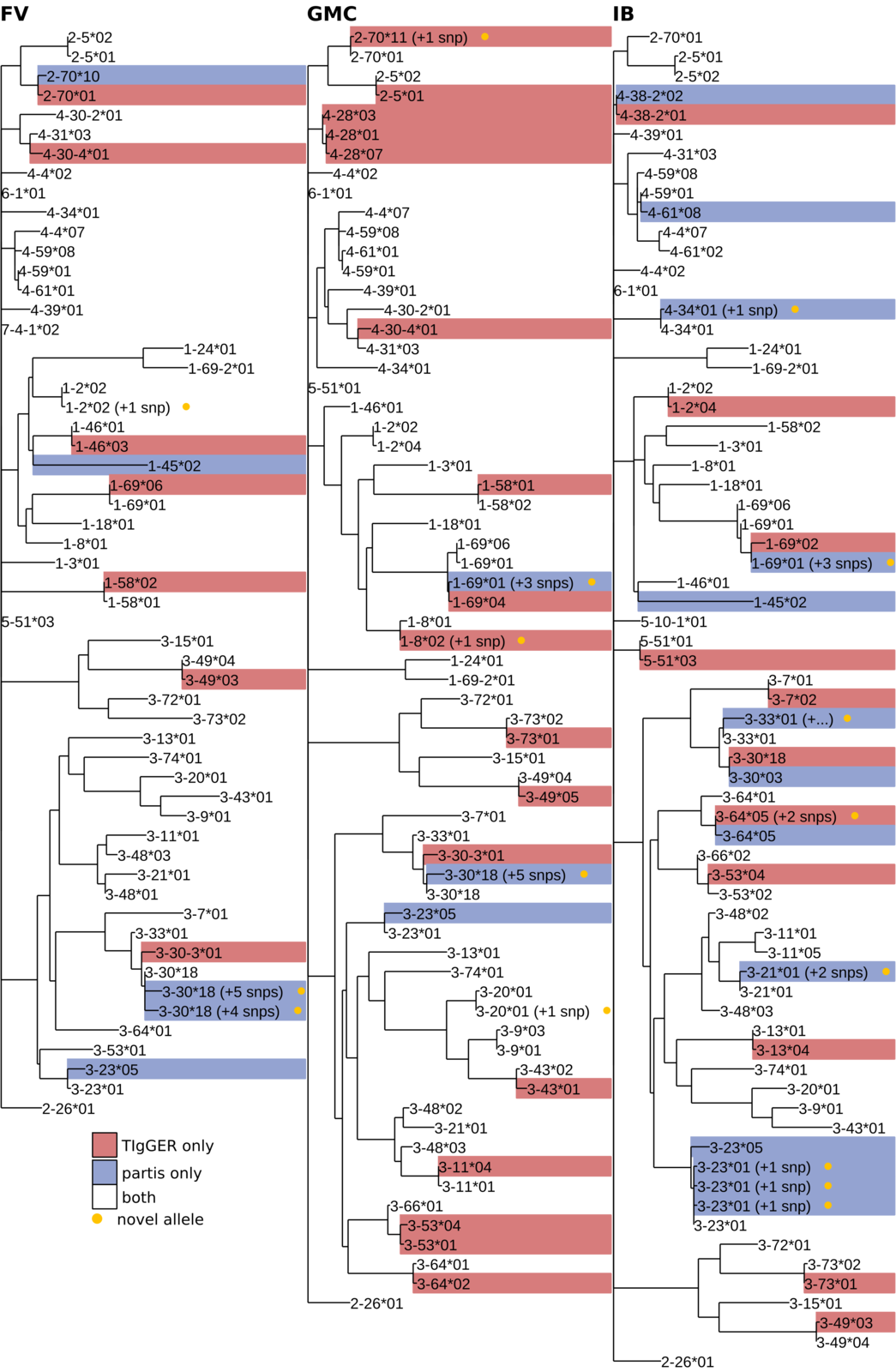

Supplement: S5 Fig — (TIFF) [file pcbi.1007133.s005.tiff]

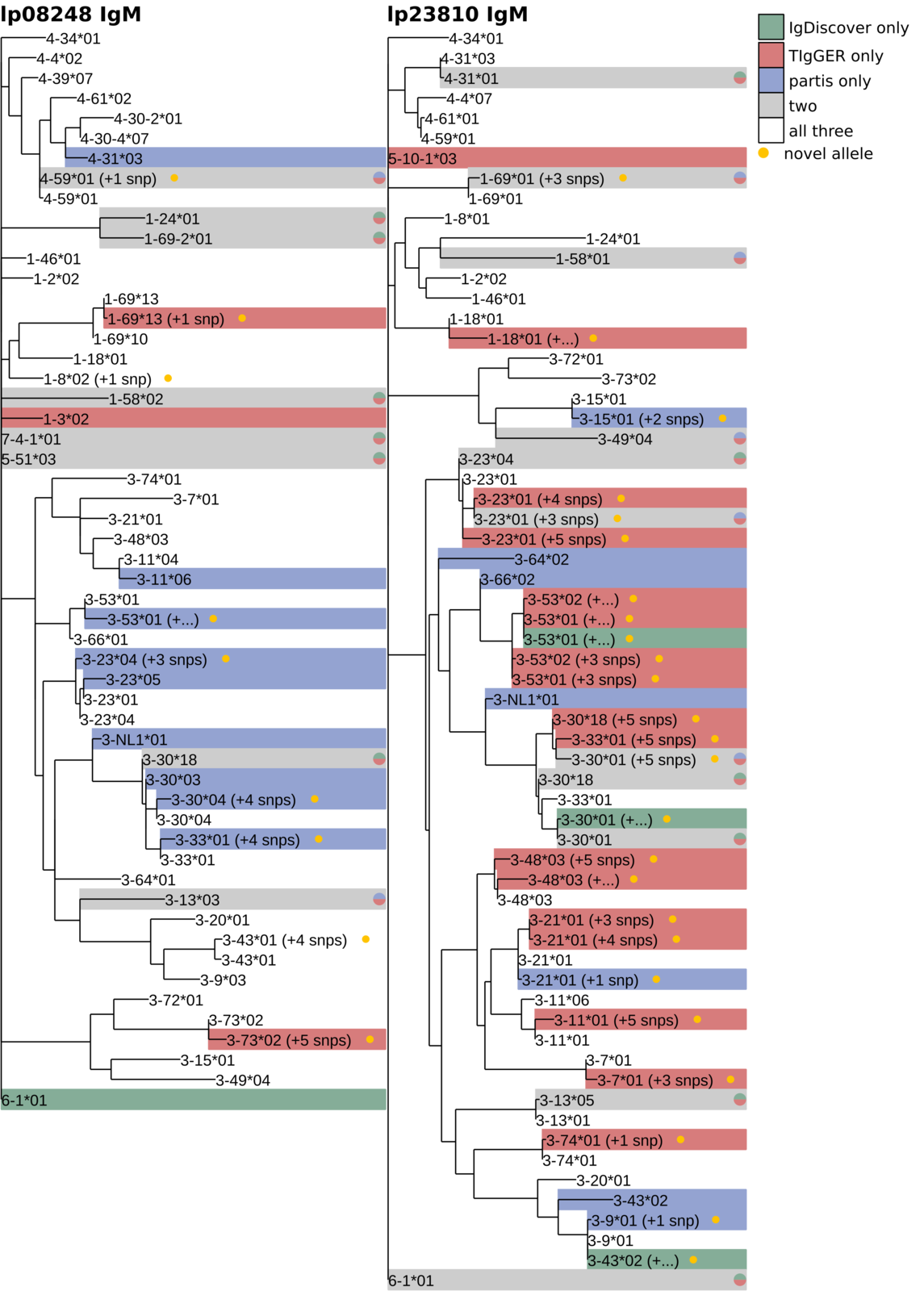

Supplement: S6 Fig — (TIFF) [file pcbi.1007133.s006.tiff]

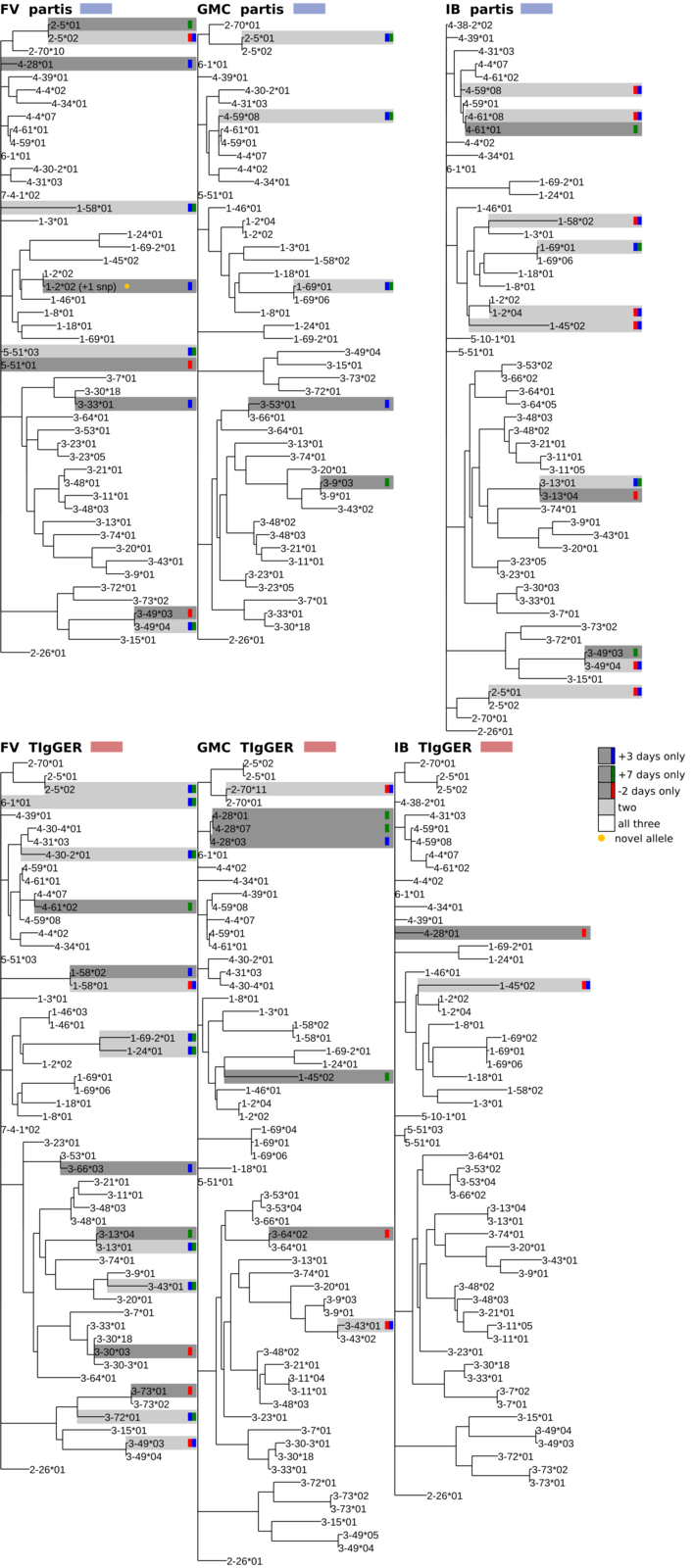

Supplement: S7 Fig — (TIFF) [file pcbi.1007133.s007.tiff]

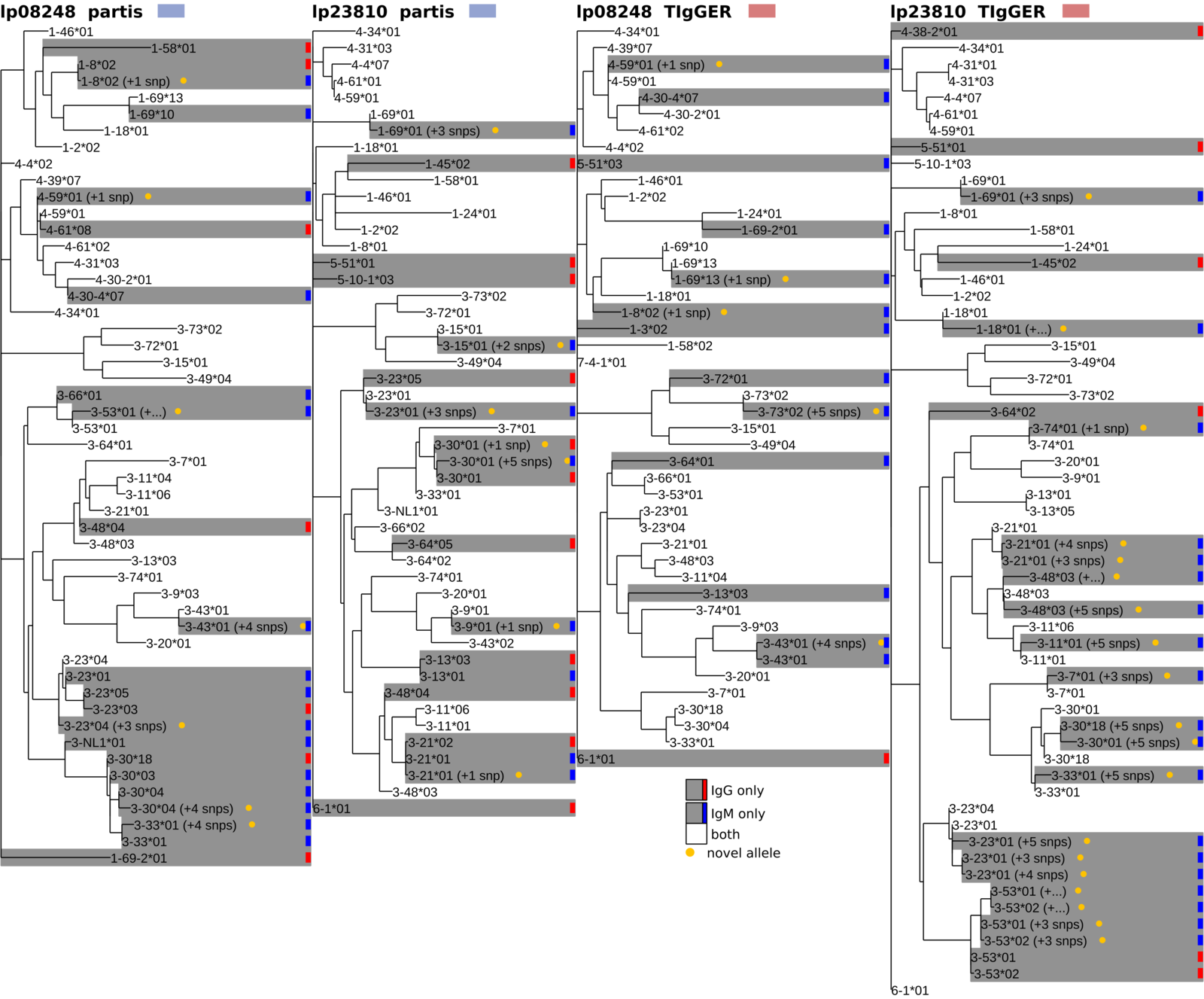

Supplement: S8 Fig — (TIFF) [file pcbi.1007133.s008.tiff]

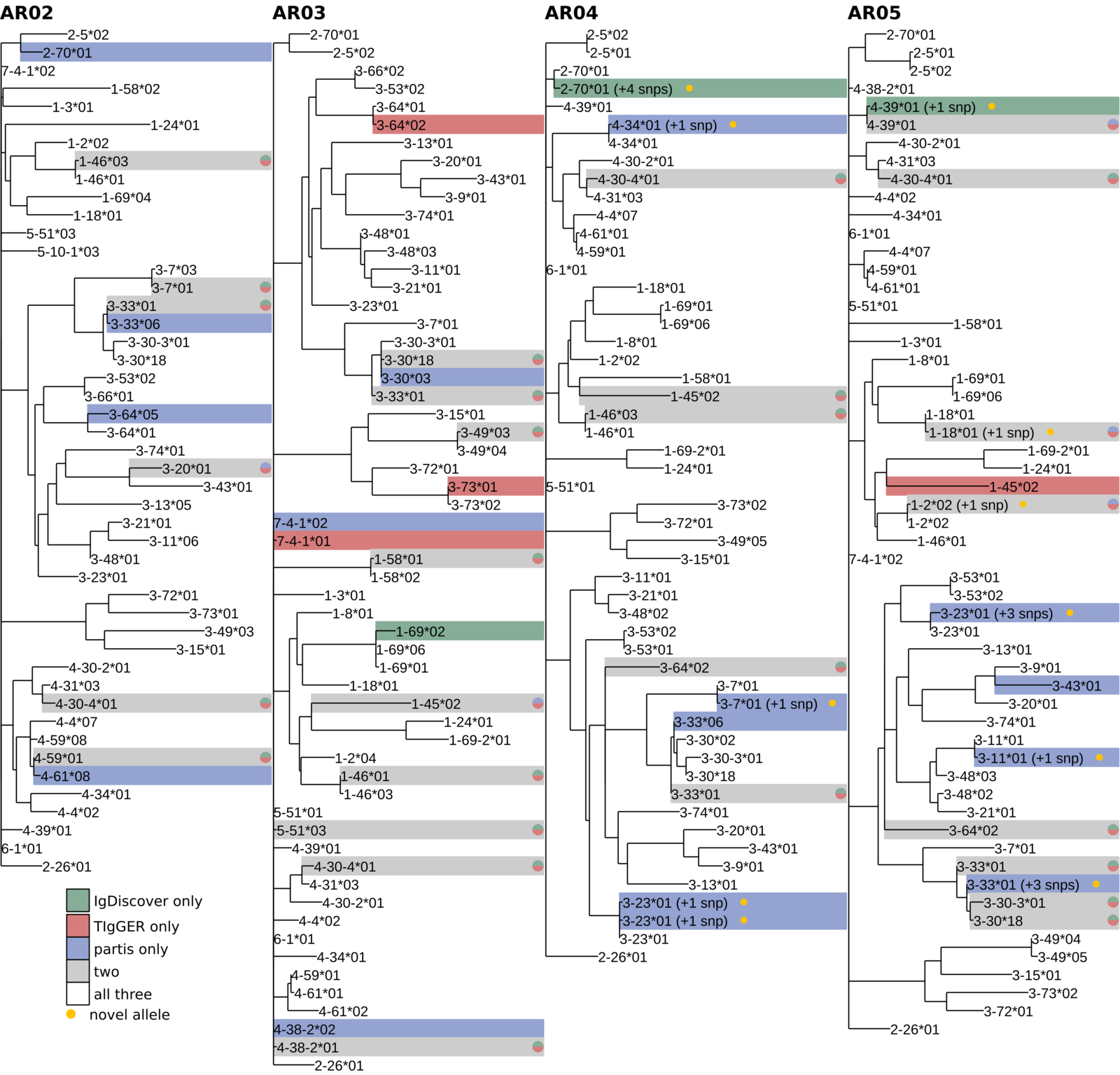

Supplement: S9 Fig — (TIFF) [file pcbi.1007133.s009.tiff]

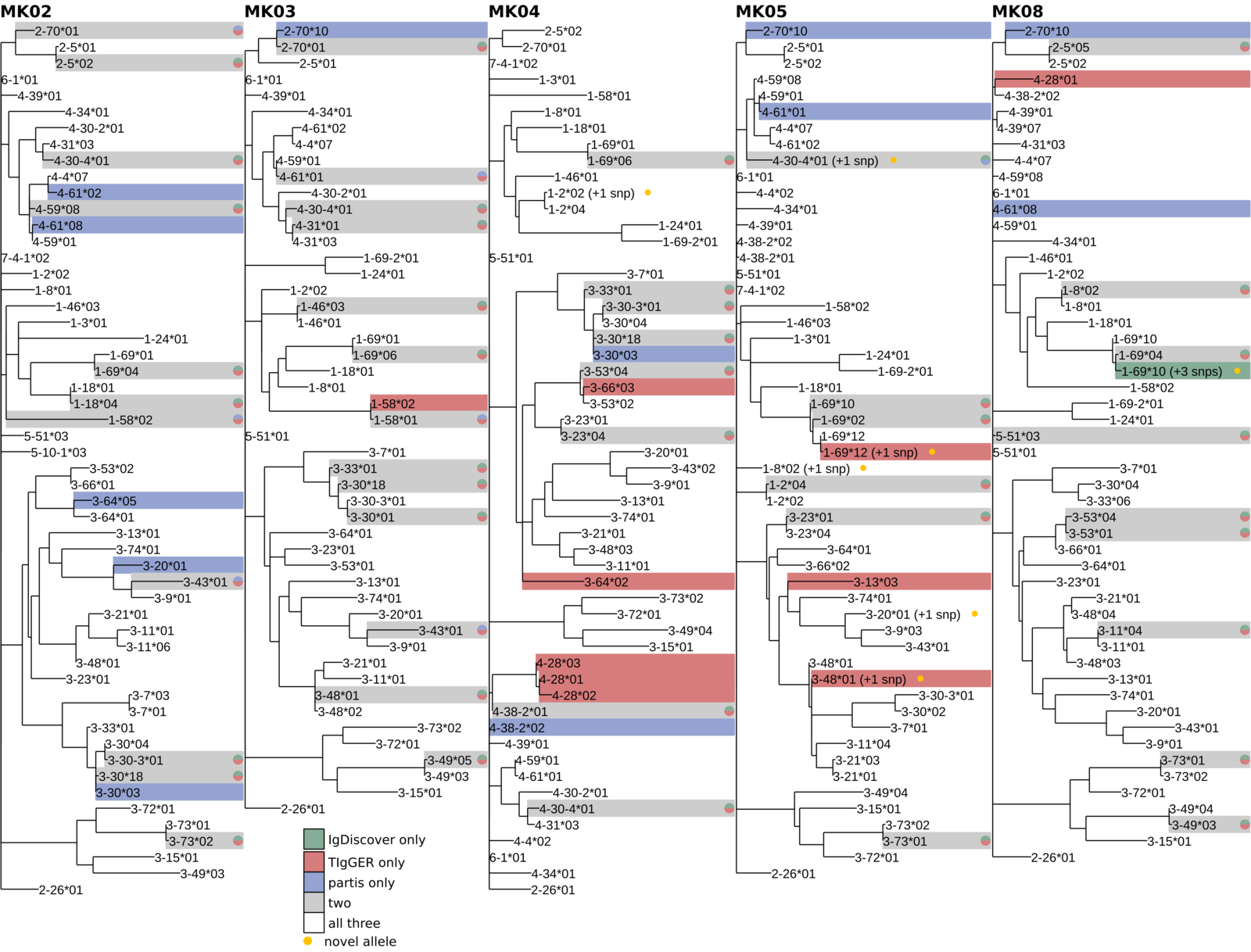

Supplement: S10 Fig — (TIFF) [file pcbi.1007133.s010.tiff]

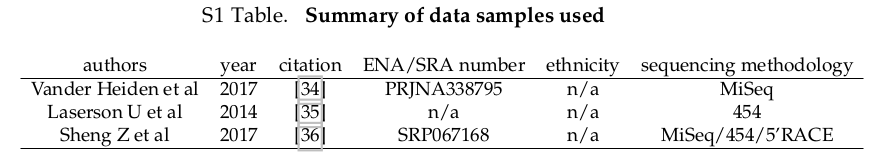

Supplement: S1 Table — (TIFF) [file pcbi.1007133.s011.tiff]

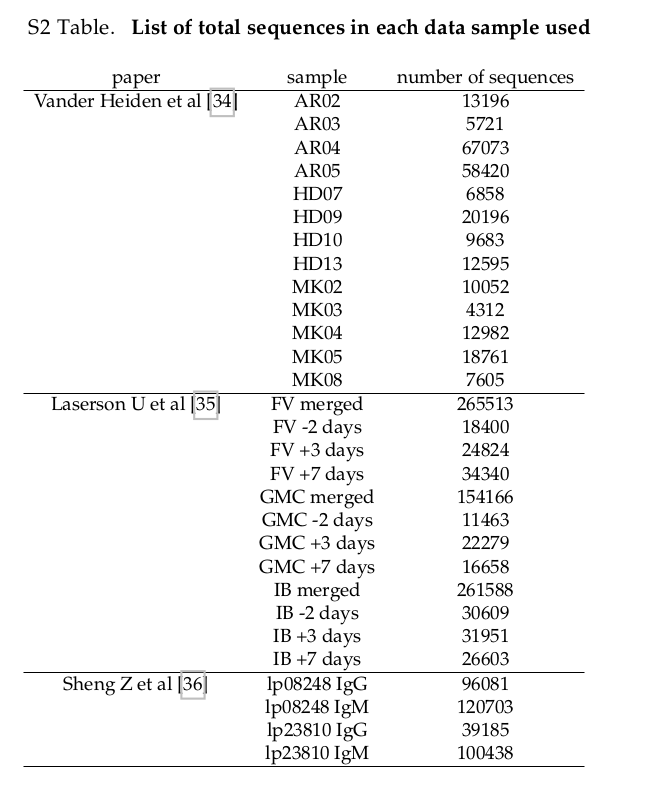

Supplement: S2 Table — (TIFF) [file pcbi.1007133.s012.tiff]
